# *Escherichia coli* displays a conserved membrane proteomic response to a range of alcohols

**DOI:** 10.1186/s13068-023-02401-4

**Published:** 2023-10-03

**Authors:** Oishi Sen, Jamie Hinks, Qifeng Lin, Qingsong Lin, Staffan Kjelleberg, Scott A. Rice, Thomas Seviour

**Affiliations:** 1 Singapore Centre for Environmental Life Sciences Engineering, Nanyang Technological University, Singapore, Singapore; 2 School of Biological Sciences, Nanyang Technological University, Singapore, Singapore; 3 Department of Biological Sciences, Faculty of Science, National University of Singapore, Singapore, Singapore; 4 School of Biological, Earth and Environmental Sciences, University of New South Wales, Sydney, 2052 Australia; 5 The Australian Institute for Microbiology and Immunology, University of Technology Sydney, Sydney, 2007 Australia; 6 CSIRO, Agriculture and Food, Westmead and Microbiomes for One Systems Health, Sydney, Australia; 7 WATEC Aarhus University Centre for Water Technology, Universitetsbyen 36, Bldg 1783, 8000 Aarhus, Denmark

**Keywords:** Bioalcohol, Bacterial stress response, *E. coli* membrane proteome, OmpC

## Abstract

**Background:**

Alcohol is a good and environment-friendly fuel that can be microbially produced, capable of eliminating many of the limitations of the present-day fossil fuels. However, the inherent toxic nature of alcohols to the microbial cells leads to end-product inhibition that limits large-scale alcohol production by fermentation. Fundamental knowledge about the stress responses of microorganisms to alcohols would greatly facilitate to improve the microbial alcohol tolerance. The current study elucidates and compares the changes in the membrane proteome of *Escherichia coli* in response to a range of alcohols.

**Results:**

Although alcohol toxicity increased exponentially with alcohol chain length (2–6 carbon), similar stress responses were observed in the inner and outer membrane proteome of *E. coli* in the presence of 2-, 4- and 6-carbon alcohols at the MIC_50_. This pertains to: (1) increased levels of inner membrane transporters for uptake of energy-producing metabolites, (2) reduced levels of non-essential proteins, associated with anaerobic, carbon starvation and osmotic stress, for energy conservation, (3) increased levels of murein degrading enzymes (MltA, EmtA, MliC and DigH) promoting cell elongation and 4) reduced levels of most outer membrane β-barrel proteins (LptD, FadL, LamB, TolC and BamA). Major outer membrane β-barrel protein OmpC, which is known to contribute to ethanol tolerance and membrane integrity, was notably reduced by alcohol stress. While LPS is important for OmpC trimerisation, LPS release by EDTA did not lower OmpC levels. This suggests that LPS release, which is reported under alcohol stress, does not contribute to the reduced levels of OmpC in the presence of alcohol.

**Conclusions:**

Since alcohol primarily targets the integrity of the membrane, maintenance of outer membrane OmpC levels in the presence of alcohol might help in the survival of *E. coli* to higher alcohol concentrations. The study provides important information about the membrane protein responses of *E. coli* to a range of alcohols, which can be used to develop targeted strategies for increased microbial alcohol tolerance and hence bioalcohol production.

**Supplementary Information:**

The online version contains supplementary material available at 10.1186/s13068-023-02401-4.

## Introduction

Microbial alcohol production, especially of alcohols with higher numbers of carbon atoms, is potentially an environmentally friendly alternative energy source to non-renewable fossil fuels [1–5]. However, alcohol toxicity is a limiting factor to the wide-spread production and use of bioalcohol. Accordingly, there is an increased interest in resolving the mechanisms of alcohol toxicity in microorganisms and hence develop more alcohol-tolerant microbes by, for example, genetic engineering approaches. In this regard, *Escherichia coli* is of special interest, since it is genetically tractable and the most well-understood bacterial species that is widely considered for commercial biofuel production [6, 7]. Therefore, it is a good model system for studying how microbial cells respond to the toxic effects of alcohols and whether the stress responses vary with an increase in alcohol chain length. *E. coli* has been engineered to produce several alcohols, such as ethanol [8–13], butanol [14–16], isopropanol [17], hexanol [18] and octanol [19]. The production of ethanol by *E. coli* is preferable as it can metabolize a range of substrates, including the sugars derived from lignocellulosic biomasses readily available from agricultural wastes [20]. It can also be engineered to produce higher alcohol titres than native producers, as demonstrated for isopropanol [17]. Furthermore, long chain, non-natural alcohols, such as hexanol and octanol, which have a higher energy content and are more environmentally friendly [1, 2], have been produced biologically in genetically engineered *E. coli* [18, 19, 21].

Various approaches have been applied to understand alcohol stress responses in *E. coli*, including transcriptomics [22, 23] and proteomics [24, 25], with a particular focus on ethanol and butanol stress response. Ethanol and butanol elicit changes in the RNA expression or levels of *E. coli* stress proteins [23–25]. Ethanol exposure increases the amount of various heat shock and general stress response proteins [24], while butanol exposure increases the expression of various oxidative, cell envelope and heat stress genes [25]. The amount of alcohol dehydrogenase YqhD increases following exposure to ethanol [24] and butanol [25]. Increased RNA expression or abundance of various inner membrane proteins was also reported for ethanol and butanol. These included subunits of efflux pumps, which export toxic compounds from the cells, as well as electron transport chain and metabolite transporter proteins, which help to meet the energy shortage arising under alcohol stress ([24, 25], Additional file 1: Table S1). Similarly, many genes associated with increased butanol tolerance in *E. coli,* encode membrane proteins, such as antiporters, efflux pumps, membrane lipoproteins, amino acid and sugar transporters [26].

**Fig. 1:**
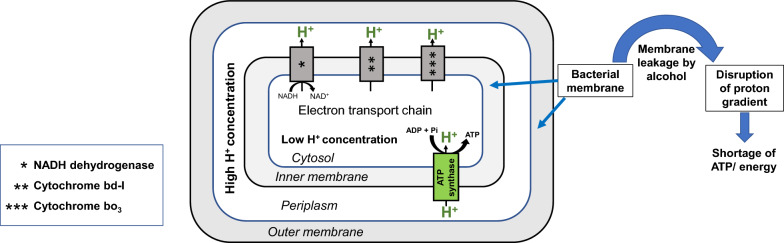
Effect of alcohol on the *E. coli* cells. Increased membrane permeability due to alcohol leads to ATP shortage or energy limitation [29, 79, 80]

Alcohol increases membrane fluidity or leakage in bacterial cells [27, 28]. Alcohols such as butanol affects the functional and structural properties of membrane and leads to increased permeability to ions and protons, thereby disrupting the proton gradient across the membrane [29]. The proton motive force (PMF) across the bacterial membrane is necessary for ATP generation [30]. Therefore, alcohol stress leads to ATP shortage or energy limitation in the bacterial cells (Fig. 1). Thus, maintenance of membrane integrity is crucial for bacteria to tolerate alcohols. In Gram-negative bacteria, such as *E. coli*, outer membrane proteins play an important role in maintaining the integrity, stability, and rigidity of the bacterial membrane. OmpA, which is one of the most abundant outer membrane proteins, supports the bacterial cell wall by interacting with the peptidoglycan [31, 32]. OmpC, another abundant outer membrane protein, maintains lipid asymmetry by transporting lipids from the outer- to the inner-membrane during the stationary phase, which in turn contributes to the integrity of the outer membrane [33, 34]. When OmpC is absent, phospholipids accumulate in the outer membrane’s outer leaflet during stationary phase, thus disrupting the outer membrane lipid asymmetry [33]. OmpC also influences the function and integrity of the outer membrane by other mechanisms that have not yet been fully accounted for [33]. LptD is an outer membrane β-barrel protein, which facilitates the transportation of LPS to the outer leaflet of the outer membrane [35], thereby helping in the maintenance of the asymmetric organization of outer membrane lipids.

Despite the importance of outer membrane proteins, there has been a greater focus on cytosolic and inner membrane protein responses to alcohol in proteomic and transcriptomic studies. Nonetheless, the amount of OmpC was found to increase in response to ethanol by 2-D gel electrophoresis and western blot analysis [36]. In contrast, it was reduced in the cell membranes of *E. coli* exposed to butanol [37]. This suggests that alcohols of different chain length may elicit different responses concerning the levels of outer membrane proteins, although it is not understood why this might be the case. (Additional file 1: Table S2) summarizes reported examples of changes in the level of various outer membrane proteins under ethanol and butanol stress. It is not understood whether similar responses will be observed for longer chain alcohols such as hexanol or whether the effects of alcohols correlate with chain length. This study, therefore, aimed to resolve membrane protein responses by *E. coli* exposed to a range of alcohols to characterize the membrane proteomic response of *E. coli* under alcohol stress.

## Results and discussion

### Glucose supplementation increases the MIC_50_ of alcohols

Alcohol stress leads to an increased abundance of sugar and amino acid transporters [24, 25], reflecting the increase in the passage of energy-producing compounds into *E. coli*. This is believed to compensate for energy limitation arising under alcohol stress (Fig. 1). Moreover, glucose supplementation increases the tolerance of *Pseudomonas putida* to butanol [38] and we postulated that higher alcohol tolerance could be similarly achieved for *E. coli* with glucose supplementation. Studies on bacterial stress responses are mostly carried out on bacterial samples exposed to the inhibiting compound at concentrations that lead to ~ 50% growth inhibition (MIC_50_) [25, 36]. Higher alcohol tolerance would increase the MIC_50_ of alcohols, allow the membrane protein responses of *E. coli* to be determined under higher alcohol concentrations, and thus provide greater resolution of the alcohol effect on the *E. coli* cell membrane.

**Fig. 2:**
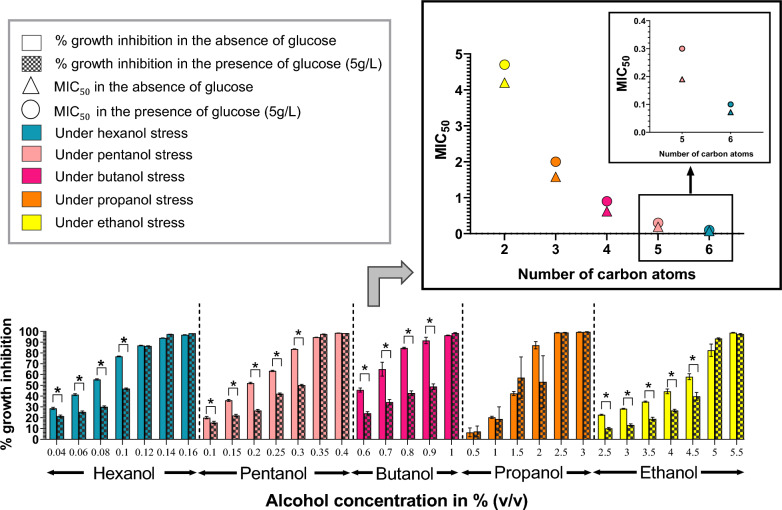
MIC_50_ of alcohols increases in the presence of glucose. The % growth inhibition with increasing concentration of hexanol, pentanol, butanol, propanol and ethanol, in the absence and presence of 5 g/L of glucose. Growth was normalised to the control cultures without alcohol. Error bars denote standard deviations of three biological replicates. The *P* value of the difference between the % growth inhibition of *E. coli* in the presence and absence of glucose is determined by unpaired *t* test. *P* value < 0.05 is considered significant and marked as *. The shift in the MIC_50_ values (determined in Additional file 2: Figure S1) for each alcohol in the presence of 5 g/L of glucose in comparison with the MIC_50_ in the absence of glucose is also shown

In the current study, *E. coli* cultures maintained higher cell densities at the same concentration of alcohol in the presence of glucose and glucose supplementation, therefore, resulted in an increase in the MIC_50_ values of the corresponding alcohols (Fig. 2, Additional file 2: Figure S1). For example, at 0.9% butanol, the growth inhibition of *E. coli* was significantly reduced from 92.7% to 48.8% when the growth medium was supplemented with 5 g/L glucose, and the MIC_50_ of butanol increased from 0.62% to 0.9% in the presence of glucose. The growth inhibition of *E. coli* by propanol, though similar to other alcohols in the absence of glucose, is different from other alcohols in the presence of glucose. Glucose was only protective at 2% propanol, where the growth inhibition was reduced from 87% to 53%. Propanol showed a similar amount of growth inhibition at concentrations of 1.5% and 2% in the presence of glucose (Additional file 2: Figure S1). The higher concentration, i.e., 2%, was selected as the MIC_50_ value of propanol as well as the propanol concentration to be used for the study. Glucose is readily used to generate energy and supplementation of the growth media with glucose compensates for the energy shortage arising from the presence of alcohol (Fig. 1). Hence, glucose supplementation is a useful method for studying the effect of alcohol on the *E. coli* membrane proteome, as it removes the interferences from stress responses to lack of energy sources under alcohol stress.

### Changes in the inner membrane proteome of *E. coli* in the presence of alcohol

#### i. Alcohol stress results in increased levels of inner membrane proteins associated with the transport of energy compounds

**Fig. 3:**
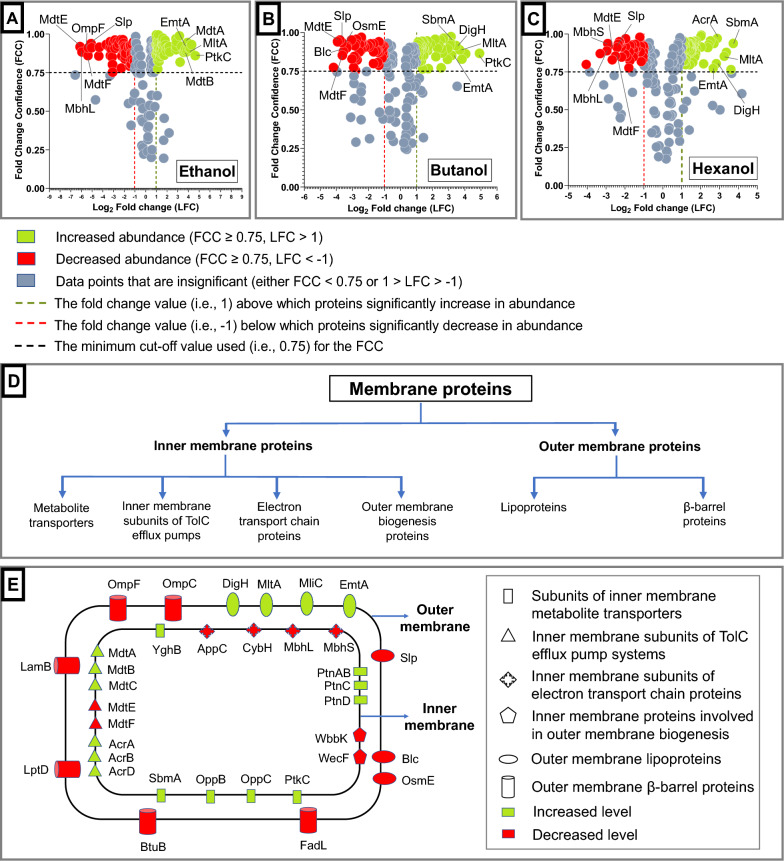
Changes in the membrane proteome of *E. coli* under alcohol stress. Volcano plots showing the changes in membrane protein levels through log_2_ fold change (LFC), under during **A** ethanol, **B** butanol and **C** hexanol stress, and the reliability of the predicted fold change values through fold change confidence (FCC). FCC > 0.75 can be considered as equivalent to *p* value < 0.05. Among the membrane proteins discussed in this study, the top five membrane proteins showing the maximum increase or decrease in their levels under ethanol, butanol or hexanol stress, are labelled in the volcano plots. **D** Types of membrane proteins discussed in this study. **E** A summary of changes in the inner and outer membrane proteome of *E. coli* under alcohol stress

**Fig. 4:**
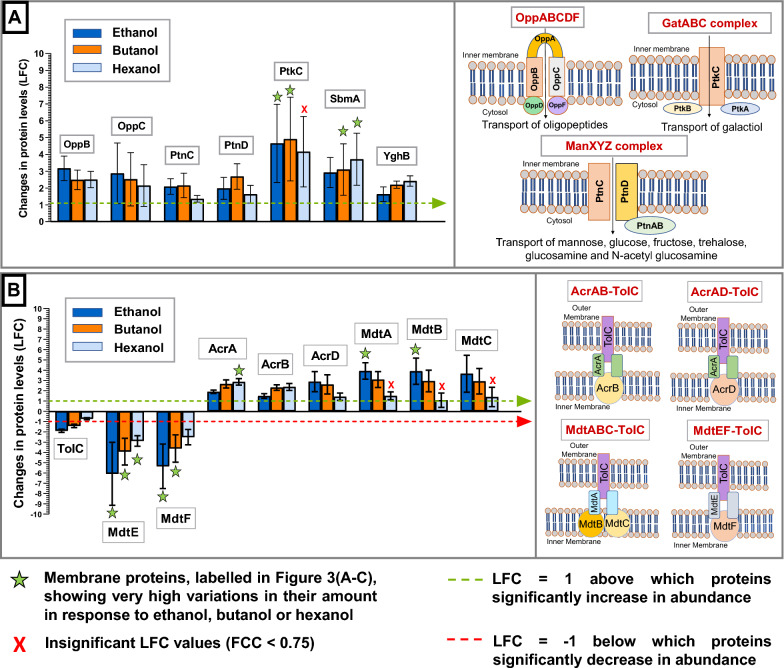
Changes in the inner membrane proteome of *E. coli* under alcohol stress (I). A comparison of the changes in the abundance of **A** subunits of inner membrane transporters, **B** inner membrane subunits of the electron transport chain under conditions of ethanol (blue), butanol (orange) and hexanol (light blue) stress. Error bars denote the standard deviations across the peptides tested with the three biological replicates. Diagrams of the inner membrane protein subunits are made using information in (Additional file 1: Table S3)

**Fig. 5:**
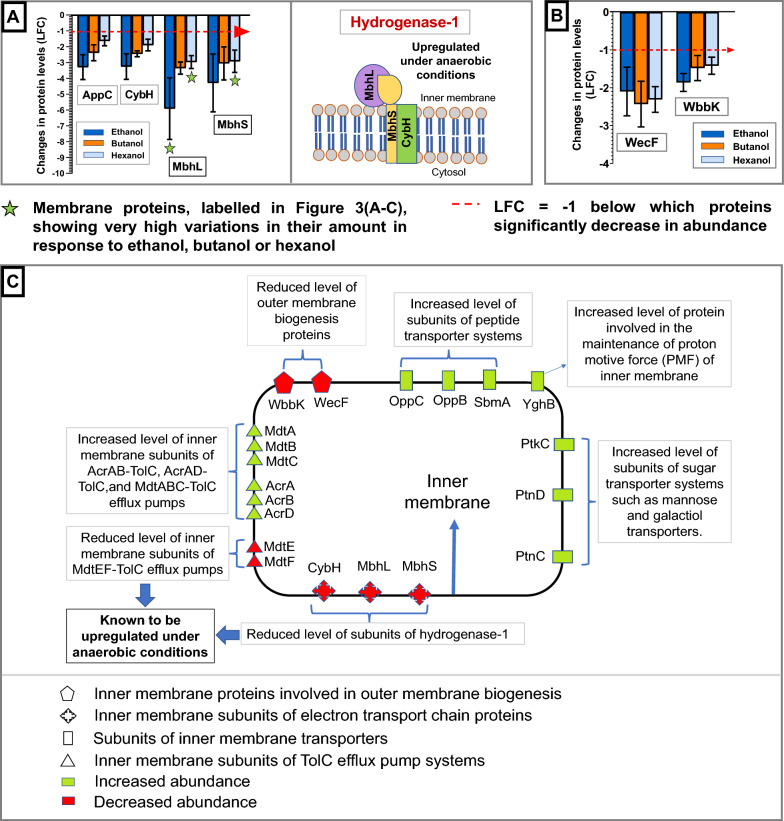
Changes in the inner membrane proteome of *E. coli* under alcohol stress (II). A comparison of the changes in the abundance of **A** inner membrane subunits of the electron transport chain and **B** inner membrane proteins involved in outer membrane biogenesis under conditions of ethanol (blue), butanol (orange) and hexanol (light blue) stress. Error bars denote the standard deviations across the peptides tested with the three biological replicates. Diagrams of the inner membrane protein subunits are made using information in (Additional file 1: Table S3). **C** A summary of changes in the inner membrane proteome of *E. coli* under alcohol stress

Sequential window acquisition of all theoretical spectra–mass spectrometry (SWATH–MS) [39], a label-free quantitative proteomics approach with high data reproducibility and accuracy, was used to compare the changes in the membrane proteome under different stress conditions. Two hundred and six membrane proteins were quantified by SWATH–MS analysis of membrane protein extracts from *E. coli* grown in the presence of ethanol, butanol and hexanol at the MIC_50_ (Fig. 3A–C). The different categories of membrane proteins discussed in this study are presented in Fig. 3D. Fold change > 2 or log_2_ fold change (LFC) > 1, representing the proteins that have at least doubled in their quantities under alcohol stress, were considered as proteins with increased abundance. Fold change < 0.5 or LFC < − 1, representing the protein populations that have reduced to at least half their quantities under alcohol stress compared to the control condition, were considered as the proteins with decreased abundance. Fold change confidence (FCC) is indicative of the reliability or the reproducibility of the reported fold change. An FCC value greater than 0.75 for the corresponding LFC value was considered equivalent to a *p* value lower than 0.05 (i.e., significant). Therefore, only LFC values with FCC > 0.75 were considered in this study. A significant increase in abundance or log_2_ fold change (LFC) was observed for various inner membrane energy-producing metabolite transporters upon exposure to all three alcohols. Specifically, subunits of the oligopeptide transport system OppABCDF (OppC and OppD), antimicrobial peptide transporter (SbmA) and sugar transport systems, such as mannose-specific enzyme II ManXYZ complex (PtnC and PtnD), galactitol-specific enzyme II GatABC complex (PtkC), were all more abundant under alcohol stress (Figs. 3E, 4A and 5C, Additional file 1: Table S3). Among the membrane proteins discussed in this study, PtkC (LFC > 4.6) showed the maximum increase in abundance in the presence of ethanol and butanol, while SbmA (LFC ≃ 3.71) showed the maximum increase in the presence of hexanol (Fig. 3A–C, green stars in Fig. 4A). Under butanol stress, SbmA also showed a very high increase in abundance.

These observations are consistent with previous studies demonstrating that alcohol exposure increases the levels of various sugar and oligopeptide transporter subunits [24, 25]. Increased amounts of OppA, OppD, OppF, PtnAB and PtnC under butanol stress [25], as well as PtnAB and PtnD under ethanol stress [24], have been reported for *E. coli*. Here, we demonstrate that the same applies for hexanol stress. An increase in the levels of these sugar and peptide transporters (Fig. 4A), known to import various energy-producing substrates into the cytosol (Additional file 1: Table S3), possibly helps to elevate the supply of energy sources under alcohol stress, since alcohol leads to energy shortage (Fig. 1). Additionally, we report an increased level of membrane transporter, YghB (LFC > 1.6), in the presence of ethanol, butanol and hexanol, which is involved in maintaining the proton motive force (PMF) of the inner membrane [40, Additional file 1: Table S3] following membrane leakage due to alcohol exposure.

#### ii. Alcohol stress elicits an inverse response to anaerobic stress regarding the abundance of inner membrane subunits of efflux pumps and electron transport proteins

Significant increases in the levels of inner membrane subunits of TolC-associated efflux pumps were observed, including MdtA (LFC > 3), MdtB (LFC > 2.9), MdtC (LFC > 2.9), and AcrD (LFC > 2.5) in the presence of ethanol and butanol, and AcrA and AcrB (LFC > 2.3) in the presence of butanol and hexanol (Figs. 3E, 4B and 5C). This is in agreement with a study reporting an upregulation of *acrB* in the presence of butanol [25]. However, the levels of subunits MdtE and MdtF were significantly reduced in the presence of ethanol, butanol and hexanol (LFC < − 2.5). MdtA (LFC ≃ 3.92) and MdtB (LFC ≃ 3.9) under ethanol stress, and AcrA (LFC ≃ 2.87) under hexanol stress, showed a very high increase in abundance among the membrane proteins. MdtE (− 6.09 ≤ LFC ≤ − 2.88) in the presence of each of the three alcohols and MdtF under ethanol (LFC ≃ − 5.35) and butanol (LFC ≃ − 3.62) stress, were among the membrane proteins showing very high reduction in their abundance (Fig. 3A–C, green stars in Fig. 4B).

Energy-dependent efflux pumps remove toxic organic solvents from bacteria [41]. The outer membrane protein TolC associates with a range of inner membrane subunits (AcrA, AcrB, AcrD, MdtA, MdtB, MdtC, MdtE and MdtF) to form different efflux pump systems, such as AcrAB–TolC, AcrAD–TolC, MdtABC–TolC and MdtEF–TolC [42, 43]. AcrB and MdtB have previously been linked to butanol tolerance [26]. Here, we observed a significant reduction in the abundance of MdtE and MdtF under alcohol stress (Fig. 4B). The expression of MdtEF is known to increase > 20-fold under anaerobic conditions and has been reported to play a critical role in the survival of *E. coli* during anaerobic growth [44]. The reduced level of MdtE and MdtF, which is relatively non-essential under the aerobic conditions used here, might reflect the induction of other essential proteins necessary for survival under conditions of decreased ATP generation due to alcohol (Fig. 1).

Electron transport chain protein subunits, such as MbhL, MbhS and CybH subunits of hydrogenase 1 and the AppC subunit of cytochrome bd-II were also reduced (− 1.6 ≥ LFC ≥ − 5.91) under alcohol stress (Figs. 3E, 5A, C). The extent of reduction in AppC protein levels was greater in the presence of ethanol (LFC ≃ − 3.29) compared to hexanol (LFC ≃ − 1.64). Similarly, the extent of reduction in MbhL protein levels was greater in the presence of ethanol (LFC ≃ − 5.91) compared to hexanol (LFC ≃ − 2.97). Among the membrane proteins discussed in this study, MbhL showed a very high decrease in abundance under ethanol and hexanol stress, while MbhS showed a very high decrease in abundance under hexanol stress (Fig. 3A, C, green stars in Fig. 5A). An earlier study reported an upregulation of other members of the electron transport chain, such as *nuo* operon (NADH dehydrogenase 1), *cyo* operon (cytochrome bo_3_) and *sdhABCD* (succinate dehydrogenase)*,* under butanol stress [25]. Hydrogenase 1 upregulates under anaerobic conditions [45, Additional file 1: Table S3]. Similar to the reduced amounts of MdtE and MdtF, a decreased level of MbhL, MbhS and CybH might enable the bacteria to reduce the expression of relatively less important proteins, such as hydrogenase-1 under energy-deficient conditions due to alcohol (Fig. 1). This may conserve the limited energy available to produce other important proteins that might facilitate cellular survival under alcohol stress.

#### iii. Alcohol reduces the levels of inner membrane proteins involved in outer membrane biogenesis

The level of inner membrane proteins involved in outer membrane biogenesis, such as WbbK (LFC < − 1.4) and WecF (LFC < − 2), was also reduced in the presence of ethanol, butanol and hexanol (Figs. 3E, 5B, C). WbbK contributes to LPS synthesis [46], and WecF is required for the synthesis of the cyclic form of the enterobacterial common antigen (ECA) [47]. LPS is the major component of the outer membrane of Gram-negative bacteria, which provides a permeability barrier and protection to the bacteria against hydrophobic molecules [48]. Other than LPS, ECA (cyclic) is also a component of the outer membrane and plays an important role in outer membrane integrity [49]. This suggests that alcohol might lead to impaired synthesis of LPS and ECA (cyclic), which would likely compromise the integrity and correct functioning of the outer membrane in the presence of alcohol.

### Changes in the outer membrane proteome of *E. coli* in the presence of alcohol

#### i. Alcohol stress leads to changes in the outer membrane lipoprotein content that facilitates cell elongation and energy conservation

**Fig. 6:**
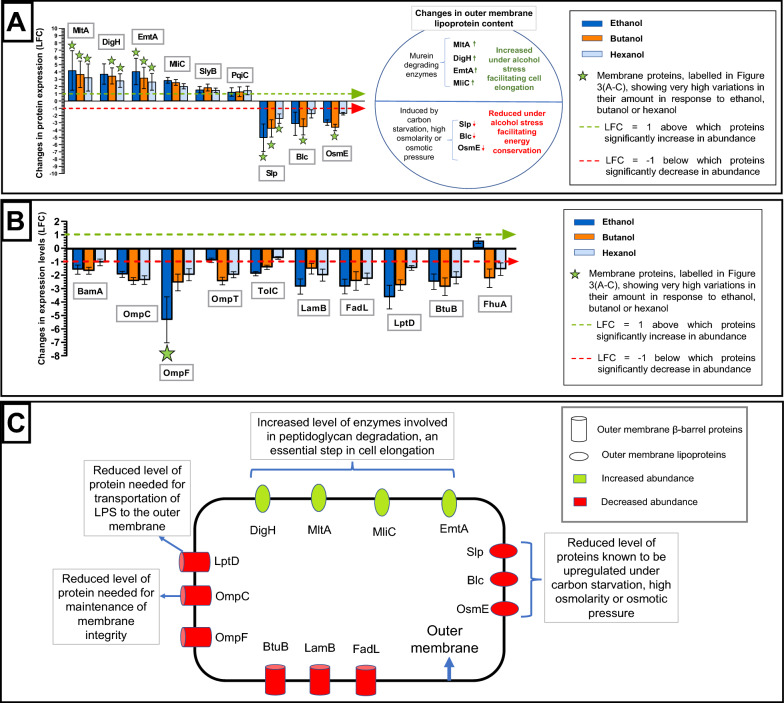
Changes in the outer membrane proteome of *E. coli* under alcohol stress. A comparison of the changes in the levels of different **A** outer membrane lipoproteins, **B** outer membrane β-barrel proteins under ethanol (blue), butanol (orange) and hexanol (light blue) stress. Here, error bars denote the standard deviation across the peptides tested with the three biological replicates, **C** A summary of changes in the outer membrane proteome of *E. coli* in the presence of alcohol. Please also see (Additional file 1: Table S4)

The levels of outer membrane lipoproteins MltA (LFC > 3.2), DigH (LFC > 2.8), EmtA (LFC > 2.6) and MliC (LFC > 2) increased under ethanol, butanol and hexanol stresses, to at least four times their original abundance in *E. coli* (Fig. 6A). Among the membrane proteins, MltA (LFC > 3.2) and EmtA (LFC > 2.6) showed a very high increase in abundance under all three alcohol stresses. Similarly, DigH (LFC > 2.8) showed a very high increase in protein levels under butanol and hexanol stress (Fig. 3A–C, green stars in Fig. 6A). Outer membrane proteins can be broadly classified into lipoproteins and β-barrel/channel proteins [50]. Outer membrane lipoproteins are proteins anchored to the outer membrane through their attached lipids. MliC plays a potential role in murein recycling after excessive hydrolysis of the murein backbone by lytic transglycosylases [51, Additional file 1: Table S4]. Similarly, MltA and EmtA have been reported to be murein hydrolyzing enzymes, while DigH is a glycosyl hydrolase that may have muramidase activity [52–55, Additional file 1: Table S4]. Since peptidoglycan (also known as murein) determines the bacterial cell shape, peptidoglycan degradation is an important step in bacterial cell elongation [56]. The elongation of bacterial cells under alcohol stress has been documented [25, 57]. Therefore, the increase in the levels of these murein degradative proteins, as seen in this current study, might facilitate the elongation of the cells under alcohol stress. Bacterial cell elongation decreases the surface area/volume ratio of the cells, which enables bacteria to sustain greater cell volume with a lower amount of membrane area exposed to toxic solvents [41]. The extent of increase in the levels of murein-degrading enzymes were similar in the presence of ethanol, butanol and hexanol. Therefore, one could postulate that the extent of cell elongation upon exposure to alcohols of different chain lengths, and at same levels of inhibition, is the same. However, cell elongation might be influenced by other factors also and these would all need to be considered when assessing factors governing cell elongation induced by alcohols of different chain lengths.

In the presence of ethanol, butanol and hexanol the level of other lipoproteins such as Slp (LFC < − 2.42), OsmE (LFC < − 1.74) and Blc (LFC <− 1.78) were reduced in the outer membrane (Fig. 6A). Slp under all three alcohol stresses, and OsmE (LFC ≃ − 3.65) and Blc (LFC ≃ − 3.56) under butanol stress (Fig. 3A–C, green stars in Fig. 6A), were among the membrane proteins showing very high reductions in their levels. OsmE was reduced more in the presence of ethanol (LFC ≃ − 2.98) and butanol (LFC ≃ − 3.65) compared to hexanol (LFC ≃ − 1.74). Slp, Blc and OsmE are induced during stationary phase and other stressful conditions, such as carbon starvation, high osmolarity and osmotic pressure, respectively [58–60, Additional file 1: Table S4]. In the current study, although the *E. coli* cells were harvested at stationary phase, they were grown in the presence of a carbon source (glucose) and under average osmolarity (Lennox LB Broth). Therefore, under the conditions used in this study, Slp, Blc and OsmE proteins were less important than other proteins necessary for *E. coli* survival under alcohol stress. Due to the limited availability of energy in the presence of alcohol (Fig. 1), there is a reduction in the levels of lipoproteins that are less essential (Slp, Blc and OsmE) and increase in the abundance of lipoproteins necessary to deal with the toxic effect of alcohol on the membrane (MltA, EmtA, DigH and MliC) (Fig. 6C). This observation is similar to the reduced levels of anaerobic proteins (MdtE, MdtF, MbhL, MbhS and CybH) observed in the presence of alcohol (Figs. 4B and 5A).

**Fig. 7:**
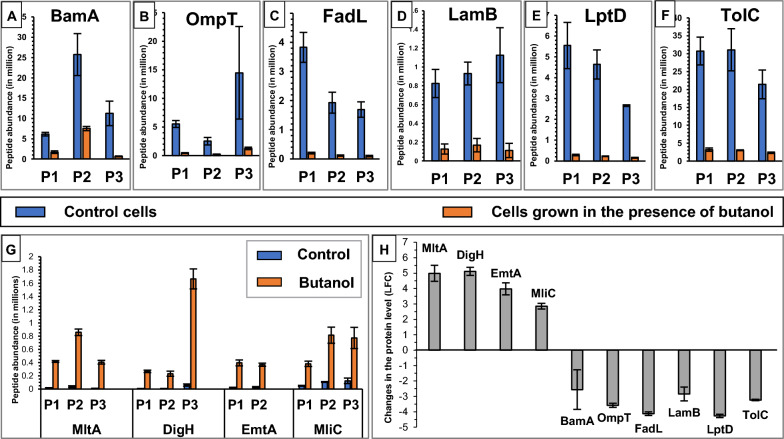
Targeted MRM^HR^ MS analysis. **A**–**F**) Comparison of the levels of BamA, OmpT, FadL, LamB, LptD and TolC outer membrane β-barrel proteins, and (**G**) MltA, DigH, EmtA and MliC outer membrane lipoproteins in control cells (blue bars) and in cells grown in the presence of butanol (orange bars) as quantified by targeted MRM^HR^ MS. The level of at least two peptides (shown as P1, P2 and P3), listed in (Additional file 1: Table S8), were detected for each protein. Error bars denote standard deviations across three biological replicates. (**H**) A comparison of the changes in the amounts of the outer membrane proteins (LFC) in the presence of butanol, calculated as an average of the peptide fold changes. Error bars denote the standard deviations in the LFC values across the peptides (≥ 2) used for each protein

Among the outer membrane lipoproteins discussed in this study, the significant increase in the levels of the murein degrading enzymes MltA, EmtA, DigH and MliC under butanol stress was validated by targeted mass spectrometry (MRM^HR^) (Fig. 7G). MltA, EmtA, DigH and MliC were selected, since the increase in the amount of these proteins could contribute to bacterial cell elongation, a phenomenon that is widely observed in the presence of alcohol [25, 57]. The level of peptides (Additional file 1: Table S8) generated from these proteins was significantly higher in the presence of butanol (orange bars) compared to the control (blue bars). Under butanol stress, MltA, DigH, EmtA and MliC proteins showed LFC values of ~ 5, 5.1, 4 and 2.9, respectively (Fig. 7H).

#### ii. Alcohol stress leads to lower levels of most outer membrane β-barrel proteins

The levels of outer membrane β-barrel proteins such as OmpC (LFC < − 1.94), OmpF (LFC < − 1.97), FadL (LFC < − 2.26), LptD (LFC < − 1.46), BtuB (LFC < − 2.19) and LamB (LFC < − 1.51) were all reduced under ethanol, butanol and hexanol stress (Figs. 3E, 6B, C). In the presence of butanol and hexanol, the level of OmpT (LFC < − 1.97) and FhuA (LFC < − 1.5) was reduced in the outer membrane (Fig. 6B), although there was no change in their levels in the presence of ethanol (1 > LFC > − 1). In the presence of ethanol and butanol, the level of BamA (LFC < − 1.58) and TolC (LFC < − 1.41) in the outer membrane was reduced although TolC showed no change in membrane protein levels under hexanol stress (1 > LFC > − 1). LptD was reduced to a greater extent in the presence of ethanol and butanol (≤ 14.8% of its original level) compared to hexanol (33.3% of its original level). OmpF showed higher reduction under ethanol (LFC ≃ − 5.33) compared to butanol (LFC ≃ − 2.55) and hexanol (LFC ≃ − 1.97), while OmpC reduced to ≤ 26% of its original level under ethanol (LFC ≃ − 1.94), butanol and hexanol (LFC ≃ − 2.4) stress. OmpF was among the membrane proteins showing a very high reduction in abundance under ethanol stress (Fig. 3A, green star in Fig. 6B). Earlier studies have reported a reduction in the levels of OmpT and OmpF on ethanol exposure [36] and a decrease in the levels of OmpC and OmpF in the presence of butanol [37], which is consistent with our findings. As OmpC maintains outer membrane integrity [33], reduction in OmpC levels would likely compromise the outer membrane barrier functions. As LptD transports LPS to the outer membrane [35], lower levels of LptD (Figs. 6B, 7E) under alcohol stress would affect the transportation of LPS to the outer membrane and, therefore, the outer membrane lipid asymmetry and integrity.

Among the outer membrane β-barrel proteins discussed in this study, the decrease in the levels of essential outer membrane β-barrel proteins, such as BamA and LptD [61], abundant outer membrane β-barrel proteins, such as protease OmpT and efflux pump subunit TolC [62–64], and substrate-specific porins, such as LamB and FadL [65, 66, Additional file 1: Table S4] under butanol stress was validated by MRM^HR^ MS. The level of peptides (Additional file 1: Table S8) generated from these proteins was significantly lower in the presence of butanol (orange bars) compared to the control (blue bars) (Fig. 7A–F, H), suggesting that alcohol stress leads to reduced levels of different types of outer membrane β-barrel proteins of diverse functions.

The membrane protein responses were very similar for the three alcohols. However, slight differences were observed in the extent of protein level changes in between the alcohols, mostly for outer membrane β-barrel proteins. Among the differentially abundant membrane proteins under alcohol stress, the changes in the protein levels were higher in the presence of short chain alcohols such as ethanol compared to medium chain alcohols, such as hexanol, for many proteins. Elucidating the regulation of these individual proteins under alcohol stress might address the reasons behind these differences. It should be noted that some of *E. coli*’s membrane proteomic stress responses to alcohol observed in the current study might vary in engineered *E. coli* strains with modified metabolic pathways.

### OmpC, but not OmpA, was reduced upon exposure to a broad range of alcohols

**Fig. 8:**
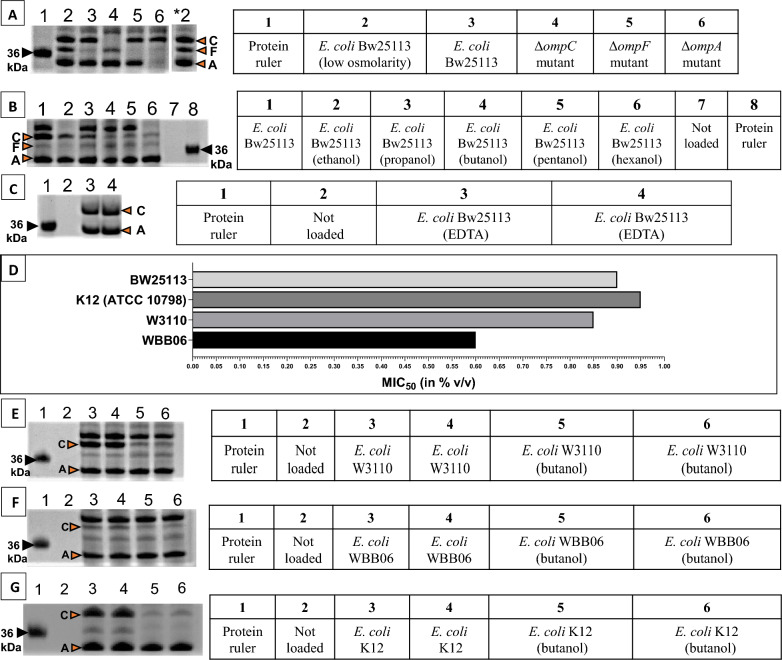
SDS–PAGE analysis. Lane 1 in (**A**–**C**, **E**–**G**) is loaded with BioRad pre-stained protein ruler (10–250 kDa) (only the part of the gel showing proteins around 36 kDa is shown here). Letters C, F and A denote proteins OmpC, OmpF and OmpA, respectively. **A** *E. coli* Bw25113 grown under low osmolarity (lane 2), *E. coli* Bw25113 (lane 3), Δ*ompC* (lane 4) Δ*ompF* (lane 5) and Δ*ompA* (lane 6) grown in LB Broth with 5 g/L glucose. *2 denotes lane 2 with the positions of the protein bands marked separately due to space constraints. **B** *E. coli* Bw25113 grown in LB Broth (with 5 g/L glucose) (lane 1), *E. coli* Bw25113 grown in LB Broth (with 5 g/L glucose) in the presence of ethanol (lane 2), propanol (lane 3), butanol (lane 4), pentanol (lane 5) and hexanol (lane 6) at concentrations leading to ~ 50% growth inhibition. **C** Two biological replicates of *E. coli* Bw25113 grown in LB Broth with 5 g/L glucose and 5 mM EDTA (lanes 3 and 4). **D** Concentration of butanol leading to ~ 50% growth inhibition (MIC_50_) in different strains of *E. coli* grown in LB Broth (with 5 g/L glucose)*.* Two biological replicates of *E. coli* (**E**) W3110, **F** WBB06 and (G) K12 (ATCC 10798) grown in the presence (lanes 5 and 6) and absence (lanes 3 and 4) of butanol. LB Broth (with 5 g/L glucose) was used as the growth media for these strains and butanol was added at concentrations leading to 50% growth inhibition, as shown in (**D**)

The observed reduction in the amount of OmpC in the *E. coli* Bw25113 membranes in the presence of alcohol was further corroborated, for ethanol, propanol, butanol, pentanol and hexanol, by SDS–PAGE analysis (Fig. 8B, lanes 2–6). OmpC, OmpF and OmpA bands (lane * 2) were assigned using deletion mutants Δ*ompC,* Δ*ompF* and Δ*ompA* (Fig. 8A, lanes 4–6). Low osmolarity growth medium (medium A) was used to stimulate a higher expression of OmpF (lane 2), relative to LB Broth with 5 g/L glucose (lane 3). While OmpA and OmpC are the most abundant outer membrane β-barrel proteins in *E. coli* (based on the number of protein molecules synthesized per generation or cell cycle; Supplementary information in [62]), OmpA was less affected by alcohol in comparison with OmpC. Alcohols with longer carbon chain lengths, such as butanol, pentanol and hexanol, elicited a greater reduction in the amount of OmpC in the outer membrane compared to short chain alcohols, such as ethanol and propanol. The effect of butanol in reducing OmpC levels was also observed in other strains of *E. coli,* such as *E. coli* K12 (ATCC 10798) and W3110 (Fig. 8E, G, lanes 5 and 6). As observed for *E. coli* Bw25113, butanol did not affect the level of OmpA in *E. coli* W3110 and K12 (ATCC 10798), compared to the large change in the level of OmpC under butanol stress.

OmpC plays an important role in alcohol tolerance. OmpC contributes to ethanol tolerance via an unknown EnvZ/OmpR-related mechanism, whereby in the absence of EnvZ, OmpR and OmpC, *E. coli* showed an increase in intracellular ethanol and decreased ethanol tolerance [36]. This is supported by another study reporting strong inhibition of cell growth under ethanol stress in *ompC*-deleted strains, compared to the wild type [32]. Higher amounts of OmpC have also been described in the cell membrane of isobutanol-adapted *E. coli* strains in the presence of isobutanol, compared to the wild type [67]. Other reports described a slight increase in butanol tolerance of *E. coli* following an overexpression of *ompC* [68, 69]. Hence, an understanding of OmpC regulation under alcohol stress is important. Lower levels of OmpC under alcohol stress would also affect the outer membrane structural integrity, as OmpC is involved in the functioning and integrity of the outer membrane [33]. Since OmpC is known to play an important role in alcohol tolerance, prevention of reduction of OmpC levels under alcohol stress might facilitate improved alcohol tolerance in the bacteria. Hence, factors contributing to OmpC reduction in the outer membrane under alcohol stress need to be identified.

### LPS release does not contribute to lower amount of OmpC in *E. coli* under alcohol stress

OmpC levels in the membrane of *E. coli* WBB06 were much lower (Fig. 8F, lanes 3 and 4) than the parent strain *E. coli* W3110 (Fig. 8E, lanes 3 and 4). In contrast to OmpC, the amount of OmpA in the cell membrane was relatively unchanged in WBB06 compared to the parent strain. *E. coli* WBB06 has mutations in LPS synthesis (*waaF)* and activity (*waaC*) genes compared to its parent strain *E. coli* W3110 [70]. This suggests that impaired LPS synthesis/activity might contribute to reduced levels of OmpC in the cell membrane, as observed here. This is consistent with reports showing that the presence of defective LPS or compounds that inhibit LPS synthesis reduce OmpC but not OmpA levels [71, 72]. One explanation for this difference could be that OmpC trimerisation, prior to the insertion of OmpC into the outer membrane, is impaired by the lack of functional LPS, since LPS is required to assemble OmpC into trimers [73, 74]. No such requirement applies to OmpA [74], as OmpA is a monomer.

It was observed that WBB06 was also the most sensitive strain to butanol toxicity among different strains of *E. coli,* such as *E. coli* W3110, K12 (ATCC 10798), WBB06 and Bw25113 (Fig. 8D), suggesting the importance of LPS in alcohol tolerance. An earlier study showed *ompC* gene transcription being upregulated despite lower levels of OmpC protein under butanol stress [37], indicating the presence of a post transcriptional or post translational regulatory mechanism that controls the levels of OmpC under alcohol stress. LPS is released from the outer membrane of *E. coli* upon butanol exposure [57], which may negatively impact the OmpC trimerisation and assembly processes after translation and prior to the insertion of OmpC in the outer membrane due to the scarcity of LPS in the outer membrane. OmpA levels would not be substantially affected by LPS shortage [74]. This could account for the lower levels of OmpC present under alcohol stress, despite little change in OmpA levels (Fig. 8B, E, G). Since LPS is important for the trimerisation of OmpC before its insertion in the outer membrane, we hypothesised that removing LPS from the outer membrane might also induce a reduction in OmpC levels in the outer membrane. Exposure of *E. coli* cells to EDTA also releases LPS [57]. In the current study, *E. coli* cells grown in the presence of 5 mM EDTA resulted in about 50% growth inhibition when the media was supplemented with 5 g/L of glucose. *E. coli* cells grown in the presence of 0.9% butanol resulted in about 50% growth inhibition when the media was supplemented with 5 g/L of glucose (Additional file 2: Figure S1C). Exposure to 5 mM EDTA releases more LPS from *E. coli* than 0.9% butanol [48]. Growing *E. coli* Bw25113 in EDTA at MIC_50_, however, did not reduce the membrane levels of OmpC (Fig. 8C, lanes 3 and 4), unlike growth of *E. coli* in butanol at MIC_50_ (Fig. 8B, E, G). This suggests that LPS release due to alcohol stress directly does not contribute to the reduced OmpC amount observed in the *E. coli* membrane in the presence of alcohol.

There could be more than one factor contributing to the lower levels of OmpC under alcohol stress. Identification of the factors contributing to OmpC reduction under alcohol stress might also address why the extent of OmpC reduction is more pronounced in the presence of medium chain alcohols such as hexanol compared to short chain alcohols, such as ethanol. As mentioned before, alcohol exposure reduces the amount of the outer membrane β-barrel protein LptD (Figs. 6B, 7E), which is required for the transportation of LPS to the outer membrane. This might lead to reduced amounts of LPS in the outer membrane and hence, affect the OmpC trimerisation and assembly processes. Thus, one factor contributing to the sharp decline in OmpC levels compared to OmpA levels in the outer membrane, under alcohol stress, could be the reduced LPS transport.

## Conclusions

The exponential increase in toxicity with alcohol chain length suggests that alcohol toxicity is amplified by steric factors as the chain length increases (2C–6C). Nevertheless, here we report a conserved membrane proteomic response by *E. coli* to ethanol, butanol and hexanol at the MIC_50_, suggesting that strategies for increased tolerance to one alcohol might apply to other alcohols as well. We confirmed that key phenotypes, such as reduced OmpC level under alcohol stress, were also observed in a range of *E. coli* strains. Since OmpC contributes to outer membrane stabilization and alcohol tolerance, the mechanism of OmpC reduction under alcohol stress should be investigated to prevent such a response of *E. coli* to alcohol. Identifying strategies for maintaining OmpC levels in the outer membrane under alcohol stress could improve the tolerance of *E. coli* to a range of alcohols.

## Materials and methods

### Culture medium

All strains of *E. coli* (Additional file 1: Table S5) were grown in 20 mL of BD Difco™ Luria–Bertani Broth (Lennox) with the following composition: 10 g/L tryptone, 5 g/L yeast extract, 5 g/L NaCl and 5 g/L glucose. To stimulate the expression of OmpF, *E. coli* Bw25113 was grown in 20 mL of medium A with the following composition: 7 g/L nutrient broth, 1 g/L yeast extract, 2 g/L glycerol, 3.7 g/L K_2_HPO_4_ and 1.3 g/L KH_2_PO_4_ [75].

### Growth inhibition studies under alcohol stress

A comparison of the relative growth inhibition of *E. coli* at different concentrations of ethanol (C2), propanol (C3), butanol (C4), pentanol (C5) and hexanol (C6), was carried out using *E. coli* Bw25113, grown in 20 mL of BD Difco™ Luria–Bertani (Lennox) with or without 5 g/L glucose (in 50 mL Greiner bio-one centrifuge tubes) at 37 °C with constant shaking at 200 RPM. Suitable concentrations of alcohol were obtained in the growth media by adding different volumes of 100% alcohol to completely cooled media and by making the final volume to 20 mL. For example, for 0.9% (v/v) butanol, 180 μL of 100% butanol was added to completely cooled 20 mL of growth media. The optical density at 600 nm (OD_600_) of the growth medium was measured after 18 h. The OD_600_ at different concentrations of alcohol was used to compare the growth inhibition of *E. coli* due to alcohol stress in the presence and absence of glucose. The % growth inhibition at different concentrations of alcohol was calculated by the following formula:

$$\% \, growth\, inhibition = \frac{{OD_{600} \,without \,alcohol - OD_{600} \, with \,alcohol}}{{OD_{600} \,without \,alcohol}} \,X \,100$$

Three biological replicates were used for each condition. An unpaired *t* test was used to determine the statistical significance of the differences between growth conditions. A *P* value < 0.05 was considered significant. MIC_50_ is the minimum inhibitory concentration of a toxic compound required to bring about 50% growth inhibition. The MIC_50_ values of C2–C6 alcohols were determined in the presence and absence of glucose. In the presence of glucose, alcohol concentrations resulting in about (50 ± 3) % growth inhibition was considered as MIC_50_ values of C2–C6 alcohols. In the absence of glucose, the MIC_50_ values of the alcohols were determined through linear interpolation of the two data points closest to 50% growth inhibition, in a % growth inhibition vs alcohol concentration graph (Additional file 2: Figure S1), since the data points were beyond the range of (50 ± 3) % growth inhibition for some alcohols.

### Extraction of whole membranes of *E. coli*

The alcohol concentration corresponding to MIC_50_, in the presence of glucose (5 g/L), was added to the growth media, while the control was grown without any alcohol. The cultures were grown for 18 h at 37 °C with constant shaking at 200 RPM. Then, the cell membranes of the *E. coli* cells were isolated by the method described in [67] with modifications. The bacterial cells were separated from the culture media by centrifugation at 4000 *xg* for 10 min using an Eppendorf Centrifuge 5810 R. The cells were then washed with phosphate-buffered saline (PBS) three times followed by centrifugation. The cell pellets were frozen at − 20 °C for 24 h and thawed at 0 °C in lysis buffer (10 mM Na_2_HPO_4_–NaOH buffer of pH 7.2) along with the DNase and protease inhibitor PMSF (phenylmethylsulfonyl fluoride). The cells were lysed using an EmulsiFlex™-C3 (AVESTIN, Canada) high-pressure homogenizer. The intact cells were removed by centrifugation at 1500xg for 20 min. The supernatant was subjected to ultracentrifugation at 100,000xg for 1 h, using Beckman Coulter Optima XPN-100 Ultracentrifuge, to isolate the cell membranes. The pelleted cell membrane isolates were then washed with lysis buffer to remove cytosolic protein contaminants. The cell membrane pellet was then dissolved in 60 µL of 100 mM triethylammonium bicarbonate–sodium deoxycholate–urea buffer (8 M Urea, 1% sodium deoxycholate and 100 mM triethylammonium bicarbonate) and kept at 4 °C overnight.

### Determination of protein concentration by BCA assay

Bovine serum albumin (BSA) stock solution (2 mg/mL) was used to prepare the standard curve (2, 1.5, 1, 0.75, 0.50, 0.25, 0.125, 0.025 and 0 mg/mL). The protein content of all of the membrane extracts were determined using the Pierce™ BCA protein assay kit. The protein concentration for each sample was normalized to the lowest sample concentration prior to SDS–PAGE, SWATH–MS and targeted mass spectrometry to ensure that each sample had the same quantity of protein.

### SDS–PAGE analysis

2X Laemmli sample buffer (125 mM Tris–HCl pH 6.8, 0.05% (w/v) bromophenol blue, 4% SDS and 20% glycerol) was added to the membrane extracts in a 1:1 ratio and heated to 95 °C for 5–10 min. The samples were then loaded (5 µL), along with prestained protein ladder from Bio-Rad (10–250 kDa), onto a 5 M urea-10% polyacrylamide gel and SDS–PAGE was performed at 80 V for 2.5 h. The gel was then stained with Coomassie brilliant blue staining solution for 20 min followed by destaining overnight. The next day, the gel image was captured using Molecular Imager Gel Doc XR + (Bio-Rad).

### SWATH–MS analysis

All LCMS analyses were performed by Protein and Proteomics Centre, Department of Biological Sciences, National University of Singapore. In this study, four different conditions with biological triplicates each were used:

1. *E. coli* grown without alcohol (condition 1)—control condition
2. *E. coli* grown with ethanol (condition 2)—stress condition
3. *E. coli* grown with butanol (condition 3)—stress condition
4. *E. coli* grown with hexanol (condition 4)—stress condition

The 12 samples of *E. coli* membrane fractions were processed using the S-Trap micro-column (Protifi) according to the manufacturer’s recommendations to generate the peptides to be analyzed by LC–MS. Reversed phase (RP) liquid chromatographic separation of the peptides were performed on the nanoLC425 system (Eksigent) using a C18 ProteCol 300 μm × 10 mm trap column (Trajan Scientific and Medical) and a ChromXP-C18-CL 75 μm × 150 mm analytical column (Eksigent). The RP solvent A (2% acetonitrile, 0.1% formic acid) and solvent B (98% acetonitrile, 0.1% formic acid) were used. The peptides were eluted using a two-step gradient as shown in the (Additional file 1: Table S6). MS analysis of the eluted peptides were performed in TripleTOF 6600 system (SCIEX). For reference spectral library generation, three technical replicate injections of the pooled 12 samples were analyzed in IDA (information-dependent acquisition) mode. Precursor ions were selected from 400 to 1600 *m/z* with 250 ms accumulation time. For MS/MS fragmentation, a maximum of 50 precursors were selected with dynamic exclusion for 15 s. The MS/MS fragmentation spectra were collected in high sensitivity mode with 50 ms accumulation time across 100–1800 *m/z* mass range and rolling collision energy enabled.

SWATH–MS mode was used for SWATH data acquisition. Precursor ion data were collected from 400 to 1600 *m/z* mass range with 50 ms accumulation time. A 100 SWATH variable window setup across 400–1200 *m/z* mass range (Additional file 1: Table S7) was used with a window overlap of 1 Da and a minimum window width of 4 Da. Rolling collision energy was enabled for each window with 5 eV spread. Fragment ion spectra was collected in high sensitivity mode across a mass range of 100–1800 *m/z* and accumulation time 30 ms.

The reference spectral library was generated by a combined search of three technical replicates using the ProteinPilot 5.02 software (SCIEX). The spectra were identified by searching against UniProt *E. coli* (strain K12) reference proteome (UP000000625, 2020 July release, 4391 entries), spiked with common contaminant proteins (cRAP) using the “thorough search” mode in the Paragon search engine (v5.0.0.0). The following parameters were specified: cysteine alkylation using methyl methanethiosulfonate, common biological modifications and detected protein threshold at 0.05. False discovery rate (FDR) analysis was automatically performed against a decoy database comprising reverse protein sequences generated from the input database.

SWATH raw files were analyzed against the reference ion library using the OneOmics workflow hosted on SCIEX Cloud OS platform, as described in [76], with some modifications. The peak area extraction parameters were: 75 ppm ion library tolerance, 10 min extracted ion chromatogram (XIC) extraction window, considering only peptides with at least 99% confidence and less than 1% FDR, and excluding shared peptides. The change in the abundance of every protein under the stress conditions compared to the control condition was represented by fold change. The fold change for every protein was calculated by the following formula:

$$Fold \,change = \frac{{Protein \,abundance\, under \,condition \,2,\, 3 \,or\, 4 \,\left( {stress\, condition} \right)}}{{Protein \,abundance\, under \,condition \,1\, \left( {control\, condition} \right)}}$$

The relative abundance of the membrane proteins detected by SWATH–MS were calculated by utilizing the normalized peak areas of each protein obtained through SWATH–MS data processing. The fold change values were converted to log_2_ fold change (LFC) values. Fold change confidence (FCC), a value calculated from the peptide variance and the peptide signal quality values [77], is an indication of the reproducibility of the reported fold change Briefly, peptide fold changes are first calculated using weighted fragment ion ratios between all replicates in the experimental group against the control group. Peptide fold change confidences are also determined using averaged transition-level reproducibility values weighted by median peptide signal-to-noise and normalization metrics. Subsequently, protein level fold changes and confidences are then calculated for two possible directions: increased or decreased. The median ratio of all peptides reporting one direction (e.g., increased) is used to determine the protein fold change. The FCC is then computed as a function of the number of peptides used and their reproducibility metrics, as well as weighted signal-to-noise metrics. The same process is repeated for peptides in the opposite direction, and the final reported confidence is the direction with the highest confidence. A fold change confidence value of > 0.75 has been determined to be roughly equivalent to a *p* value of < 0.05 [78].

### Data visualization

Global membrane proteome responses of *E. coli* Bw25113 under stress conditions in comparison with control were visualized as volcano plots representing fold change confidence (statistical significance) with respect to fold changes of all the detected membrane proteins. Only the membrane protein fold changes were visualized. Cytosolic and periplasmic proteins were considered as contaminants.

### Targeted mass spectrometry validation

Targeted MS validation of selected proteins was performed using high-resolution MRM (MRM^HR^). Representative peptides were selected from the SWATH MS data based on proteotypicity, i.e., unique to protein and good MS detectability. Proteomics sample processing and LCMS analysis were performed as described above, except the following. The RP solvent A was 0.1% formic acid, while solvent B was 0.1% formic acid in acetonitrile. The peptides were chromatographically separated using a Acclaim PepMap100 C18 3 μm 100 Å, 75 μm × 250 mm analytical column (Thermo Scientific). The MRM^HR^ MS analysis consisted of a TOF–MS scan across 400–1600 m*/z* with 50 ms accumulation time and 33 product ion scans across 100–1800 m*/z* with 85 ms accumulation time. The product ion scan parameters are detailed in (Additional file 1: Table S8). MRM^HR^ data were processed using the Quantitation Workflow in the Analytics Module in SCIEX OS 2.1.6. Peak integration was performed with the AutoPeak algorithm with the following parameters: retention time half window 60 s and XIC width 0.05 Da.

### Supplementary Information

**Additional file 1: Table S1. **Inner membrane proteins with increased gene expression/protein levels under alcohol stress according to previous studies. **Table S2.** Outer membrane proteins with altered gene expression/protein levels under alcohol stress according to previous studies. **Table S3.** Functions of the inner membrane proteins detected in the study. **Table S4.** Functions of the outer membrane proteins discussed in the study. **Table S5.** *E. coli* strains used in this study. **Table S6.** Liquid chromatography gradient profile used for peptide elution. **Table S7: **SWATH variable window setup. **Table S8.** MRM^HR^ product ion scan parameters.

**Additional file 2: Figure S1.** Comparison of the MIC_50_ values of (A) ethanol, (B) propanol, (C) butanol, (D) pentanol and (E) hexanol determined in the presence (pink bars, right hand side) and absence (brown triangles, left hand side) of glucose. In the presence of glucose (5 g/L), alcohol concentrations resulting in about (50 ± 3) % growth inhibition was considered as MIC_50_ values of respective alcohols. In the absence of glucose, the MIC_50_ values of the alcohols were determined through linear interpolation of the two data points closest to 50% growth inhibition, in a % growth inhibition vs alcohol concentration graph, since the data points were beyond the range of (50 ± 3) % growth inhibition for some alcohols.

## Data Availability

The mass spectrometry proteomics data have been deposited to the ProteomeXchange Consortium via the PRIDE [89] partner repository with the data set identifier PXD043021.

## Abbreviations

LFC: Log_2_ fold change
FCC: Fold change confidence
MIC_50_: The minimum inhibitory concentration of a toxic compound (alcohol) required to bring about 50% growth inhibition
LPS: Lipopolysaccharide
EDTA: Ethylenediaminetetraacetic acid
PMSF: Phenylmethylsulfonyl fluoride
SWATH: Sequential window acquisition of all theoretical spectra
LC: Liquid chromatography
MS: Mass spectrometry
PBS: Phosphate-buffered saline
SDS: Sodium dodecyl sulphate
PAGE: Polyacrylamide gel electrophoresis
MRM: Multiple reaction monitoring
HR: High-resolution
PMF: Proton motive force
