# Supplementary material for: Escherichia coli displays a conserved membrane proteomic response to a range of alcohols
Source: Biotechnol Biofuels Bioprod. 2023 Oct 3;16:147. doi: 10.1186/s13068-023-02401-4 (PMC10546733; doi:10.1186/s13068-023-02401-4)
Supplement: Supplementary file 1 — Additional file 1: Table S1. Inner membrane proteins with increased gene expression/protein levels under alcohol stress according to previous studies. Table S2. Outer membrane proteins with altered gene expression/protein levels under alcohol stress according to previous studies. Table S3. Functions of the inner membrane proteins detected in the study. Table S4. Functions of the outer membrane proteins discussed in the study. Table S5. E. coli strains used in this study. Table S6. Liquid chromatography gradient profile used for peptide elution. Table S7: SWATH variable window setup. Table S8. MRMHR product ion scan parameters. [file 13068_2023_2401_MOESM1_ESM.docx]

**Additional file**

**Table S1: Inner membrane proteins with increased gene expression/protein levels under alcohol stress according to previous studies.**

| Alcohol  (v/v) | Genes | Proteins | *E. coli* strain | Technique(s)  used | Section of protein complex | Function | References |
| --- | --- | --- | --- | --- | --- | --- | --- |
| 4% ethanol | *manX*, *manZ* | PtnAB,  PtnD | BW25113 | SILAC | ManXYZ complex | Transport of mannose | [24], [25], [81] |
| 0.8% butanol | *manX*,  *manY* | PtnAB,  PtnC | DH1 (ATCC 33849) | Microarray,  iTRAQ |  |  |  |
| 4% ethanol | *malE* | MalE | BW25113 | SILAC | MalEFGK complex | Transport of maltose or maltodextrin | [24], [81] |
| 0.8% butanol | *acrB* | AcrB | DH1 (ATCC 33849) | Microarray, qPCR | AcrAB-TolC efflux pump system | Expulsion of various antibiotics and toxic compounds | [25], [63] |
| 0.8% butanol | *oppA, oppD,*  *oppF* | OppA, OppD,  OppF | DH1 (ATCC 33849) | iTRAQ | OppABCF complex | Transport of oligopeptides | [25], [81] |
| 0.8% butanol | *cyoB* | CyoB | DH1 (ATCC 33849) | Microarray,  iTRAQ | Cytochrome bo3 | Member of the electron transport chain and ATP synthesis | [25], [81] |
| 0.8% butanol | *nuoB* | NuoB | DH1 (ATCC 33849) | Microarray  iTRAQ | NADH dehydrogenase 1 (NDH-1) |  | [25], [81] |
| 3.125% ethanol | *atpB* | Atp6 | K-12 K99+ | 2D gel and MALDI-TOF | ATP synthase |  | [36], [81] |

**Table S2: Outer membrane proteins with altered gene expression/protein levels under alcohol stress according to previous studies.**

| Gene/ Protein | Alcohol | Expression changes/ protein levels | *E. coli* strain | Techniques  used | References |
| --- | --- | --- | --- | --- | --- |
| *ompF* | 0.8% butanol | Decreased | DH1 (ATCC 33849) | Microarray,  qPCR | [25] |
| OmpT  OmpF  Tsx  OmpA | 3.125% ethanol | Decreased | K-12 K99+ | 2D gel and MALDI-TOF, western blot | [36] |
| OmpC | 1.5% butanol | Decreased | MG1655 | Use of OmpF deletion strain and 1D SDS-PAGE analysis | [37] |
| OmpF | 1.5% butanol | Decreased | MG1655 | Use of OmpC deleted strain and 1D SDS-PAGE analysis | [37] |
| LamB  FadL  OmpC | 3.125% ethanol | Increased | K-12 K99+ | 2D gel and MALDI-TOF, western blot | [36] |

**Table S3: Functions of the inner membrane proteins detected in the study.**

| Gene name | Protein subunits | Transport  System/ Enzyme complex | Function | Reference |
| --- | --- | --- | --- | --- |
| *oppB* | OppB | Oligopeptide transport system OppABCDF | Translocates oligopeptides across inner membrane | [82] |
| *oppC* | OppC |  |  |  |
| *manY* | PtnC | Mannose specific Enzyme II ManXYZ complex | Phosphoenolpyruvate-(PEP)-dependent-sugar phosphotransferase systems (PTS)  involved in the transport of mannose, glucose, fructose, trehalose, glucosamine and N-acetyl glucosamine | [81], [83] |
| *manZ* | PtnD |  |  |  |
| *gatC* | PtkC | Galactitol-specific Enzyme II  GatABC complex | PTS complex involved in galactitol transport | [81] |
| *sbmA* | SbmA | Peptide antibiotic transporter SbmA | Uptake of antimicrobial peptides | [81] |
| *yghB* | YghB | Membrane transporter YghB | Involved in the maintenance of proton-motive force (PMF) | [40] |
| *appC* | AppC | Cytochrome bd-II | Generates a proton motive force that can drive ATP synthesis | [80], [81] |
| *hyaA* | MbhS | Hydrogenase-1 | Synthesized during anaerobic growth, oxidizes hydrogen to protons and electrons and delivers electrons to the electron transport chain | [81], [45] |
| *hyaB* | MbhL |  |  |  |
| *hyaC* | CybH |  |  |  |

**Table S4: Functions of the outer membrane proteins discussed in the study.**

| Protein name | Function | Reference |
| --- | --- | --- |
| MltA | Murein hydrolases | [51] |
| DigH | Glycosyl hydrolase with probable muramidase activity | [52] |
| EmtA | Murein hydrolases | [53] |
| MliC | Membrane-bound lysozyme inhibitor of C-type lysozyme | [54], [55] |
| Slp | Induced by stationary phase and carbon starvation | [58] |
| Blc | Induced by high osmolarity or starvation, helps in the transport/storage of lipids for  membrane maintenance under stressful conditions | [59] |
| OsmE | Induced by osmotic pressure and stationary phase | [60] |
| BamA | A subunit of the β-barrel-assembly machinery (BAM) complex | [84] |
| OmpC | Passive diffusion of small molecules in and out of the bacterial cell | [85] |
| OmpF | Passive diffusion of small molecules in and out of the bacterial cell | [85] |
| OmpT | Membrane protease that degrades cationic antimicrobial peptides | [64] |
| LamB | Transport of maltose and maltose polymers | [65] |
| TolC | Component of multidrug AcrAB-TolC efflux pump | [63] |
| FadL | Uptake of long chain fatty acids | [66] |
| LptD | Component of LPS transport complex, which is involved in the insertion of LPS into the outer membrane | [33] |
| BtuB | Uptake of Vitamin B12 across the outer membrane | [86] |
| FhuA | Transport of ferric siderophore ferrichrome | [87] |

**Table S5: *E. coli* strains used in this study.**

| **Strains** | **Description** | **Ref/source** |
| --- | --- | --- |
| Bw25113 | Parent strain of the knock-out strains of Keio collection. | NBRP (NIG, Japan): *E. coli* [88] |
| JW2203-KC/ Δ*ompC* | *E. coli* Bw25113 with the deletion of the gene *ompC.* | NBRP (NIG, Japan): *E. coli* [88] |
| JW0912-KC/ Δ*ompF* | *E. coli* Bw25113 with the deletion of the gene *ompF.* | NBRP (NIG, Japan): *E. coli* [88] |
| JW0940-KC/ Δ*ompA* | *E. coli* Bw25113 with the deletion of the gene *ompA.* | NBRP (NIG, Japan): *E. coli* [88] |
| W3110 | Wild type strain (F−, λ−) used for butanol response studies. | [70] |
| WBB06 | W3110 mutant with a mutation of *waaC* and *waaF* genes. | [70] |
| K12 | Commonly used laboratory wild type strain (F+, λ+) of *E. coli*. | ATCC 10798 |

**Table S6: Liquid chromatography gradient profile used for peptide elution.**

| **Time** | **Flow rate (nL/min)** | **%A** | **%B** |
| --- | --- | --- | --- |
| 0 | 300 | 95 | 5 |
| 60 | 300 | 95 | 15 |
| 120 | 300 | 70 | 30 |
| 123 | 300 | 10 | 90 |
| 143 | 300 | 10 | 90 |
| 145 | 300 | 95 | 5 |
| 160 | 300 | 95 | 5 |

**Table S7: SWATH variable window setup**

| **Start mass** | **Stop mass** | **Collision energy spread** |
| --- | --- | --- |
| 399.5 | 407.1 | 5 |
| 406.1 | 414.3 | 5 |
| 413.3 | 420.9 | 5 |
| 419.9 | 427.5 | 5 |
| 426.5 | 434.1 | 5 |
| 433.1 | 440.7 | 5 |
| 439.7 | 446.7 | 5 |
| 445.7 | 452.7 | 5 |
| 451.7 | 458.7 | 5 |
| 457.7 | 464.1 | 5 |
| 463.1 | 470.1 | 5 |
| 469.1 | 475.5 | 5 |
| 474.5 | 481.5 | 5 |
| 480.5 | 486.9 | 5 |
| 485.9 | 492.9 | 5 |
| 491.9 | 498.3 | 5 |
| 497.3 | 503.7 | 5 |
| 502.7 | 509.1 | 5 |
| 508.1 | 514.5 | 5 |
| 513.5 | 519.9 | 5 |
| 518.9 | 525.3 | 5 |
| 524.3 | 530.7 | 5 |
| 529.7 | 536.1 | 5 |
| 535.1 | 541.5 | 5 |
| 540.5 | 546.3 | 5 |
| 545.3 | 551.7 | 5 |
| 550.7 | 556.5 | 5 |
| 555.5 | 561.3 | 5 |
| 560.3 | 566.1 | 5 |
| 565.1 | 570.9 | 5 |
| 569.9 | 575.1 | 5 |
| 574.1 | 579.9 | 5 |
| 578.9 | 584.1 | 5 |
| 583.1 | 588.9 | 5 |
| 587.9 | 593.1 | 5 |
| 592.1 | 597.9 | 5 |
| 596.9 | 602.1 | 5 |
| 601.1 | 606.3 | 5 |
| 605.3 | 611.1 | 5 |
| 610.1 | 615.3 | 5 |
| 614.3 | 619.5 | 5 |
| 618.5 | 623.7 | 5 |
| 622.7 | 627.9 | 5 |
| 626.9 | 632.1 | 5 |
| 631.1 | 636.3 | 5 |
| 635.3 | 640.5 | 5 |
| 639.5 | 644.7 | 5 |
| 643.7 | 648.9 | 5 |
| 647.9 | 653.7 | 5 |
| 652.7 | 658.5 | 5 |
| 657.5 | 662.7 | 5 |
| 661.7 | 667.5 | 5 |
| 666.5 | 671.7 | 5 |
| 670.7 | 676.5 | 5 |
| 675.5 | 680.7 | 5 |
| 679.7 | 685.5 | 5 |
| 684.5 | 690.3 | 5 |
| 689.3 | 695.1 | 5 |
| 694.1 | 699.9 | 5 |
| 698.9 | 704.7 | 5 |
| 703.7 | 710.1 | 5 |
| 709.1 | 714.9 | 5 |
| 713.9 | 720.3 | 5 |
| 719.3 | 725.1 | 5 |
| 724.1 | 730.5 | 5 |
| 729.5 | 735.9 | 5 |
| 734.9 | 741.3 | 5 |
| 740.3 | 746.7 | 5 |
| 745.7 | 752.7 | 5 |
| 751.7 | 758.7 | 5 |
| 757.7 | 765.3 | 5 |
| 764.3 | 771.3 | 5 |
| 770.3 | 778.5 | 5 |
| 777.5 | 785.1 | 5 |
| 784.1 | 792.3 | 5 |
| 791.3 | 800.1 | 5 |
| 799.1 | 807.9 | 5 |
| 806.9 | 815.7 | 5 |
| 814.7 | 824.1 | 5 |
| 823.1 | 832.5 | 5 |
| 831.5 | 840.3 | 5 |
| 839.3 | 848.1 | 5 |
| 847.1 | 856.5 | 5 |
| 855.5 | 864.3 | 5 |
| 863.3 | 872.7 | 5 |
| 871.7 | 881.7 | 5 |
| 880.7 | 891.3 | 5 |
| 890.3 | 900.9 | 5 |
| 899.9 | 910.5 | 5 |
| 909.5 | 922.5 | 5 |
| 921.5 | 935.1 | 5 |
| 934.1 | 948.9 | 5 |
| 947.9 | 963.9 | 5 |
| 962.9 | 979.5 | 5 |
| 978.5 | 998.1 | 5 |
| 997.1 | 1019.1 | 5 |
| 1018.1 | 1046.7 | 5 |
| 1045.7 | 1085.7 | 5 |
| 1084.7 | 1131.9 | 5 |
| 1130.9 | 1199.7 | 5 |

**Table S8: MRM^HR^ product ion scan parameters**

| **Proteins** | **Peptides** | **Precursor theoretical *m/z*** | **CE** |
| --- | --- | --- | --- |
| MltA | Peptide 1: LYGNQSNVYNAVQEWLR | 685.3412 | 30.9 |
|  | Peptide 2: QGEFQYPIYR | 650.8197 | 30.9 |
|  | Peptide 3: GQHFDIYQGIGPEAGHR | 471.2302 | 21.6 |
| DigH | Peptide 1: LGINTVFFQVKPDGTALWPSK | 773.4230 | 35.1 |
|  | Peptide 2: IGENPGYDPLQFMLDEAHKR | 583.2863 | 27.2 |
|  | Peptide 3: ILPWSDLMTGK | 630.8362 | 29.9 |
| EmtA | Peptide 1: INDLDADEFLEHVAR | 586.2882 | 26.1 |
|  | Peptide 2: AMQWMPISQK | 610.3017 | 28.9 |
| MliC | Peptide 1: MQTDTLEYQC[MSH]DEKPLTVK | 730.0039 | 33 |
|  | Peptide 2: IVLNNC[MSH]QLQNPQR | 793.4005 | 37.9 |
|  | Peptide 3: QEVSFVYDNQLLHLK | 611.6562 | 27.4 |
| BamA | Peptide 1: GLEDFYYSVGK | 639.3061 | 30.3 |
|  | Peptide 2: LAGDLETLR | 494.2771 | 23.2 |
|  | Peptide 3: AGLGYVHNSLSNMQPQVAMWR | 787.0527 | 35.8 |
| OmpT | Peptide 1: NGAGIENYNFITTAGLK | 891.9547 | 42.7 |
|  | Peptide 2: STETLSFTPDNINADISLGTLSGK | 827.7500 | 37.7 |
|  | Peptide 3: MPYIGLTGSYR | 629.3184 | 29.8 |
| FadL | Peptide 1: AYSGEGAIADDAGNVSR | 826.8792 | 39.5 |
|  | Peptide 2: TGIAFDDSPVPAQNR | 794.3917 | 37.9 |
|  | Peptide 3: INEGPYQFESEGK | 749.3464 | 35.7 |
| LamB | Peptide 1: FVVQYATDSMTSQGK | 831.3956 | 39.7 |
|  | Peptide 2: NLIEWLPGSTIWAGK | 842.9565 | 40.3 |
|  | Peptide 3: FAYNINNNGHMLR | 521.9210 | 23.1 |
| LptD | Peptide 1: TVDALGNVHYDDNQVILK | 672.0129 | 30.3 |
|  | Peptide 2: IASANQVTTGVTSR | 702.8757 | 33.4 |
|  | Peptide 3: GLSSNYGLGTQEMLR | 813.4012 | 38.9 |
| TolC | Peptide 1: YNYLINQLNIK | 698.3852 | 33.2 |
|  | Peptide 2: NNLDNAVEQLR | 643.3284 | 30.5 |
|  | Peptide 3: AQYDTVLANEVTAR | 775.8941 | 37 |
