# Supplementary material for: Escherichia coli displays a conserved membrane proteomic response to a range of alcohols
Source: Biotechnol Biofuels Bioprod. 2023 Oct 3;16:147. doi: 10.1186/s13068-023-02401-4 (PMC10546733; doi:10.1186/s13068-023-02401-4)
Supplement: Supplementary file 2 — Additional file 2: Figure S1. Comparison of the MIC50 values of (A) ethanol, (B) propanol, (C) butanol, (D) pentanol and (E) hexanol determined in the presence (pink bars, right hand side) and absence (brown triangles, left hand side) of glucose. In the presence of glucose (5 g/L), alcohol concentrations resulting in about (50 ± 3) % growth inhibition was considered as MIC50 values of respective alcohols. In the absence of glucose, the MIC50 values of the alcohols were determined through linear interpolation of the two data points closest to 50% growth inhibition, in a % growth inhibition vs alcohol concentration graph, since the data points were beyond the range of (50 ± 3) % growth inhibition for some alcohols. [file 13068_2023_2401_MOESM2_ESM.docx]

**(A)**

**(B)**

**(C)**

**(D)**

**(E)**

**Figure S1:** A comparison of the MIC_50_ values of (A) ethanol, (B) propanol, (C) butanol, (D) pentanol and (E) hexanol determined in the presence (pink bars, right hand side) and absence (brown triangles, left hand side) of glucose. In the presence of glucose, alcohol concentrations resulting in about (50 ± 3) % growth inhibition was considered as MIC_50_ values of respective alcohols. In the absence of glucose, the MIC_50_ values of the alcohols were determined through linear interpolation of the two data points closest to 50% growth inhibition, in a % growth inhibition vs alcohol concentration graph, since the data points were beyond the range of (50 ± 3) % growth inhibition for some alcohols.
